# Properties Characterization of Chemically Modified Hemp Hurds

**DOI:** 10.3390/ma7128131

**Published:** 2014-12-17

**Authors:** Nadezda Stevulova, Julia Cigasova, Adriana Estokova, Eva Terpakova, Anton Geffert, Frantisek Kacik, Eva Singovszka, Marian Holub

**Affiliations:** 1 Department of Material Engineering, Institute of Environmental Engineering, Faculty of Civil Engineering, Technical University of Kosice, Vysokoskolska 4, Kosice 042 00, Slovakia; E-Mails: julia.cigasova@tuke.sk (J.C.); adriana.estokova@tuke.sk (A.E.); eva.terpakova@tuke.sk (E.T.); eva.singovszka@tuke.sk (E.S.); marian.holub@tuke.sk (M.H.); 2 Department of Chemistry and Chemical Technologies, Faculty of Wood Sciences and Technology, Technical University in Zvolen, T. G. Masaryka 2117/24, Zvolen 960 53; Slovakia; E-Mails: geffert@tuzvo.sk (A.G.); kacik@tuzvo.sk (F.K.)

**Keywords:** hemp hurds, chemical modification, FTIR, XRD, TG/DSC, SEC

## Abstract

The effect of chemical treatment of hemp hurds slices in three solutions (EDTA (Ethylenediaminetetraacetic acid), NaOH and Ca(OH)_2_) on the properties of natural material was discussed in this paper. Changes in the morphology, chemical composition and structure as well as thermal stability of hemp hurds before and after their modification were investigated by using FTIR (Fourier transform infrared spectroscopy), XRD (X-ray powder diffraction analysis) and TG (thermogravimetry)/DSC (differential scanning calorimetry). Size exclusion chromatography (SEC) measurements were used for determination of degree of cellulose polymerization of hemp hurd samples. Chemical modification is related to the partial removal of non-cellulosic components of lignin, hemicellulose and pectin as well as waxes from the surface of hemp hurd slices. Another effect of the chemical treatment applied is connected with increasing the crystallinity index of cellulose determined by FTIR and XRD methods. Decrease in degree of cellulose polymerization and polydispersity index in chemically modified hemp hurds compared to the original sample was observed. Increase in thermal stability of treated hemp hurd was found. The most significant changes were observed in alkaline treated hemp hurds by NaOH.

## 1. Introduction

Composites derived from natural and fast renewable resources, especially cellulosic materials, are increasing in importance due to their numerous advantageous properties for application in sustainable building constructions. The composites require a strong fiber with good adhesion between matrix and fiber to enhance their final properties. Substantial research work has been conducted on fiber reinforced concrete primarily made of a mix of hydraulic cement, aggregates, water, and reinforcing fibers. Fibers used are typically synthetic industrial such as steel, polypropylene, nylon, and glass. Adding such materials to concrete should result in a randomly, discrete, and discontinuous orientation of fibers whose main function is to bridge across cracks that develop in concrete. Thus, fiber reinforced concrete is expected to satisfy the strength, ductility, and durability requirements of a high performance concrete material [1]. In the scientific sphere as well as in industrial production, the use of natural lignocellulosic fibers (such as sisal, jute, bamboo, coconut, coir, kenaf, hemp, *etc.*) as a replacement for synthetic fibers into composites has received attention in recent years. One of the aspects of using hemp products in building industry is they are more environmentally friendly in comparison to conventional materials (conventional insulating materials-polystyrene, mineral wool and materials based on cement binders and other).

Considerable efforts have been made to develop natural fiber-reinforced cement composites for affordable infrastructures. However, the long-term durability of natural fiber-reinforced composites is limited due to their high permeability and lack of resistance to crack growth, particularly fibers obtained from agricultural by-products [2,3]. A major problem in plant fibers compared to synthetic fibers is missing homogeneity of the material and hydrophility of natural fibers resulting in high moisture absorption.

**Figure 1:**
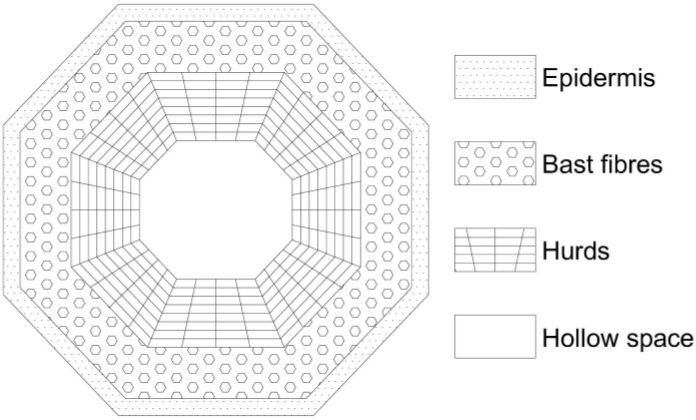
Cross-section of a hemp stem [6].

The technical hemp (Cannabis Sativa L.) is the source of two types of natural fibers: bast fibers (used mainly in the paper and textile industries) and woody core fibers—hurds. Hemp stem consists of approximately 20 wt%–40 wt% of bast fibers and 60 wt%–80 wt% of hurds [4]. The hurds consist of 40%–48% cellulose, 18%–24% hemicellulose and 21%–24% lignin. The bast fibers contain higher amounts of cellulose (57%–77%) and content of hemicellulose (9%–14%) and lignin (5%–9%) is lower compared to woody core fibers [5]. A cross section of the hemp stem shows its complex structure of different layers preset in stem (Figure 1). The outside of the stem is covered with bark, also called epidermis. Inside the hemp stems are bast fibers and hurds. The bast fibers are joined together by a middle lamella, mainly composed of pectin and arranged in bundles forming a ring around the outer part of the stem. Each fiber bundle consists of single fibers. There are two types of fibers, the useful primary fibers (5–55 mm long) and the short secondary fibers (2 mm long). The bast fibers are 10 to 100 times longer than the woody fibers in the hurds. Their diameters are approximately the same; however the cell walls of the bast fibers are 5 to 10 times thicker than those of woody fibers. The fiber content is given in relation to the weight of the whole stem [6]. In contrast to the high quality of bast fibers, the hurds is the least valuable part of the plant, chemically very close to wood.

Hemp can play a vital role in the move towards organic agriculture. This plant is easy to grow. For its growth it needs no chemical fertilizers and herbicides. Growth conditions have a huge influence on the production of plant fibers [7].

Growing interest in using hemp hurds as waste material from bast fibers production in lightweight composite preparation is recorded in recent years [8,9]. Interesting properties such as thermal, mechanical, acoustic and aseptic including low density, biodegrability, low costs and ecological suitability of this plant raw material predetermine to use it in function of organic filler in composite. In our previous papers [10,11,12], experimental study of the parameters affecting the physical and mechanical properties of hemp composite based on conventional and alternative binders was performed with emphasis on an assessment of the possibility using industrial hemp as a source of natural fibers for purpose of construction. However, high moisture sorption and heterogeneity of hemp fibers cause weak adhesion of the fibers and the matrix in composite system and a poor transfer of the applied stress between the filler and binder materials, what was confirmed in [13]. Indeed, due to the highly porous structure of hemp and its strong capillarity effects inside the tubes, hemp is able to absorb large amounts of water (up to five times its own weight). On the other hand, this hemp ability to regulate humidity inside buildings by absorbing and/or releasing water depending on air conditions cannot be neglected [14].

Many research projects have been devoted to the enhancement of the adhesion at the fibers–matrix interface by using chemical modifications of the surface of fibers the [15,16] or physical treatment of hemp fibers [17,18]. Whereas the benefits of surface treatments of natural fibers on the fiber–polymer matrix interfacial properties were intensively studied and are known, the potential positive effect of chemically treated of hemp hurds on fiber/inorganic matrix interface has not been systematically studied, especially not in the case of alternative inorganic binder such as MgO–cement. The influence of chemical modification on strength parameters as well as behavior of composites based on modified hurds with alternative binder of MgO–cement was investigated in our previous papers [19,20]. Changes in chemical composition of hemp hurds affect the properties of the resulting composite, which is produced by incorporating biomass into the composite system. Therefore, knowledge of the chemical changes in organic filler achieved by treatment is necessary for clarifying its behavior in interaction with inorganic matrix and it is very important for understanding the possible effects on final physical and mechanical properties of hardened composites based on chemically modified hemp hurds. In this article, complex study of the changes in surface morphology, chemical composition and structure of hemp hurds after chemical modification in three medium have been characterized by using of various methods (FTIR (Fourier Transform infrared spectroscopy), XRD (X-ray powder diffraction analysis) and TG (thermogravimetry)/DSC (differential scanning calorimetry) and SEC (Size exclusion chromatography) and compared to reference (unmodified) hemp hurds.

## 2. Experimental Section

### 2.1. Material

**Figure 2:**
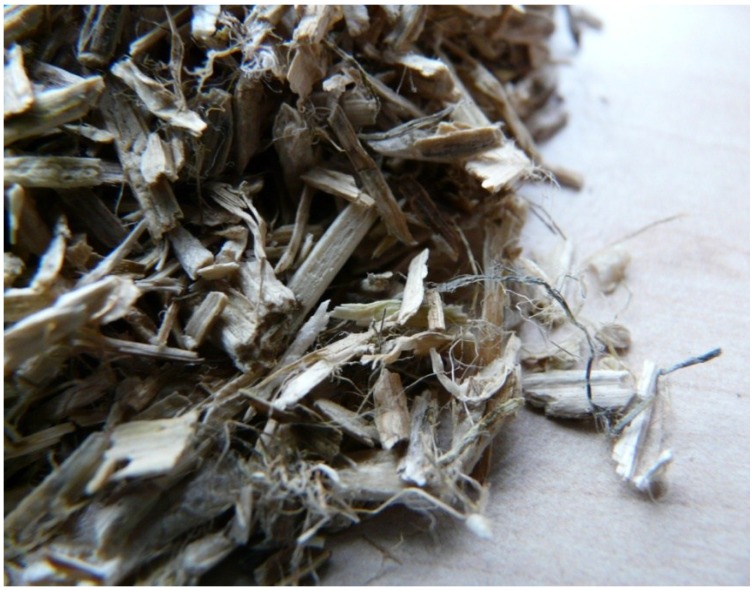
Hemp hurd slices.

In an experimental study, hemp hurds coming from the Netherlands Company Hempflax (Oude, Pekela, The Netherlands) were used. Original hemp hurds slices had wide particle size distribution of particles (8–0.063 mm). This material consisted of a large majority of hemp hurds over hemp bast fibers, and it also contained fine dust particles originating from the manufacturing grinding process (Figure 2).

The mass yield of fractions polydisperse sample of hemp hurds is given in Table 1. The mean particle length of hemp hurds was 1.94 mm. Density of hemp material was 117.5 kg·m^−3^. The average moisture content of hemp material determined by weighing of hemp sample before and after drying for 24 h at 105 °C was found 10.78 wt%. The chemical composition of hemp material is shown in Table 2.

**Table 1:** Granulometric analysis of hemp hurds.

Fraction (mm)	Mass yield (wt%)
8–2	46.4
<2	53.6

**Table 2:** Chemical composition of hemp hurd slices (original sample).

Hemp hurds component	Content (%)
Toluen-ethanol extract	3.57
Holocellulose	77.28
Cellulose	44.5
Hemicellulose	32.78
Lignin	21.03
Ash	3.04

A milled and oven-dried sample was used for the determination of chemical composition of hemp hurds. Extractives were determined in a Soxhlet apparatus (Kavalier Glass, Sázava, Czech Republic) with a mixture of ethanol and toluene (2:1) for 8 h according to the American Society for Testing and Materials [21]. Total content of polysaccharides (*i.e.*, holocellulose) was determined using the method of Wise *et al*. [22]. Cellulose content was determined by the Seifert method [23]. A mixture of acetylacetone, dioxane, and hydrochloric acid (6:2:1.5) under reflux for 30 min was used for delignification of samples. The content of hemicelluloses was determined as the difference between holocellulose and cellulose. The content of acid-insoluble (Klason) lignin was determined according to the U.S. Department of Energy, National Renewable Energy Laboratory analytical procedure [24]. The samples were hydrolyzed in a two-stage process. In the first stage, 72% (*w*/*w*) H_2_SO_4_ at a temperature of 30 °C was used for 2 h, and in the second stage, the samples were refluxed after dilution to 4% (*w*/*w*) H_2_SO_4_ for 4 h. Total ash content (mineral substances) was determined according to the U.S. Department of Energy, National Renewable Energy Laboratory analytical procedure [25].

#### Chemical Modification of Hemp Hurds

Chemical modification of hemp hurd slices was carried out in three chemical environments: in a saturated solution of ethylenediaminetetraacetic acid (EDTA), calcium hydroxide and in solution of sodium hydroxide (Table 3).

**Table 3:** Used chemicals for chemical modification of hemp hurds.

Chemical	Formula	Producer	Purity
Ethylenediaminetetraacetic acid	C_10_H_16_O_8_N_2_	GAVAX s.r.o., Vranov n/Toplou, Slovakia	per analysis
Calcium hydroxide	Ca(OH)_2_	ROTH GmbH, Karlsruhe, Germany	≥96%, pulverized
Sodium hydroxide	NaOH	CHEMAPOL, Bratislava, Slovakia	per analysis

Chemical modification of hemp hurds was performed under conditions according to literature data [26]. Following procedures of hemp hurds treatment were used:

Treatment by EDTA: Hurds were immersed during 3 h in a saturated solution of EDTA (5 g/L).

Treatment by Ca(OH)_2_: In order to saturate fibers with calcium ions, fibers were placed in a saturated lime solution ([Ca^+2^] = 2.1 × 10^−2^ M) for 48 h.

Alkalization with NaOH: The dried hemp hurds were soaked in 1.6 M NaOH solution during 48 h and then neutralized in a 1 vol% acetic acid.

In order to ensure removal of chemicals excess used for treatment of hemp hurds, all prepared samples were washed with water until the pH value 7 and subsequently dried in an oven at 70 °C until a constant weight.

### 2.2. Methods

#### 2.2.1. Optical Microscopy

The changes in structure of hemp fiber bundles were examined by optical (light) microscopy Micrographs were obtained by using of stereo microscope with highest optical performance (Nikon SMZ 1500, NIKON, Tokyo, Japan).

#### 2.2.2. Water Absorption Measurement

The hydrophilic nature of hemp fibers is a major problem for their use as reinforcement in composites. Therefore it is relevant to detect water absorption behavior of chemically modified hemp hurds and to compare to unmodified sample. Water absorption tests of the reference sample and modified hemp hurds dried at 60 °C in air oven for 24 h were carried out by their immersing in de-ionized water bath (polyethylene closed container) at 23 °C for one hour. The percentage of absorbed water in hemp hurd samples after immersion was calculated from the weight difference between the samples full immersed in water and the dry samples.

#### 2.2.3. FTIR Spectroscopy

For providing a more complete picture of molecular structure of hemp hurds before and after chemical treatment, FT infrared spectroscopy (FTIR) was used in conjunction with other techniques as X-ray powder diffraction analysis (XRD), thermal analysis TG/DSC and gel chromatography.

FTIR measurements were carried out on Bruker Alpha Platinum-ATR spectrometer (BRUKER OPTICS, Ettingen, Germany). A total of 24 scans were performed on each sample in the range of 400–4000 cm^−1^.

For the determination of crystalline cellulose content (crystallinity index—CI) in hemp hurds, FTIR spectroscopy and XRD analysis were used. Intensities (*I*) of some bands in IR spectra have been found to be sensitive to variations in cellulose crystallinity. The baseline corrected absorption peaks at 1423 and 896 cm^−1^ correspond to the sensitive frequencies of crystalline and amorphous cellulose structures, respectively. The intensities ratio of these peaks (*I*_1423_ and *I*_896_) has been most frequently used to measure relative cellulose crystallinity [27]. The CI_FTIR_ in hemp hurds was calculated using the Equation (1):

CI_FTIR_ (%) = (*I*_1423_ − *I*_896_/*I*_1423_) × 100 (1)

#### 2.2.4. XRD Analysis

The crystalline phase of cellulose in hemp samples was identified with a diffractometer, the Bruker D2 Phaser (BRUKER AXS, Karlsruhe, Germany) in Bragg-Brentano geometry (configuration Theta-2Theta), using Ni-filtered CuKα radiation (λ = 0.15406 nm) generated at 30 kV and 10 mA. Scanning conditions were identical for all samples and the X-ray diffractograms were recorded through 2θ = 10° to 40°, a step size of 0.04° (2θ) and step time of 100 s. The XRD patterns were processed using the software Diffrac.EVA v.2.1.

**Figure 3:**
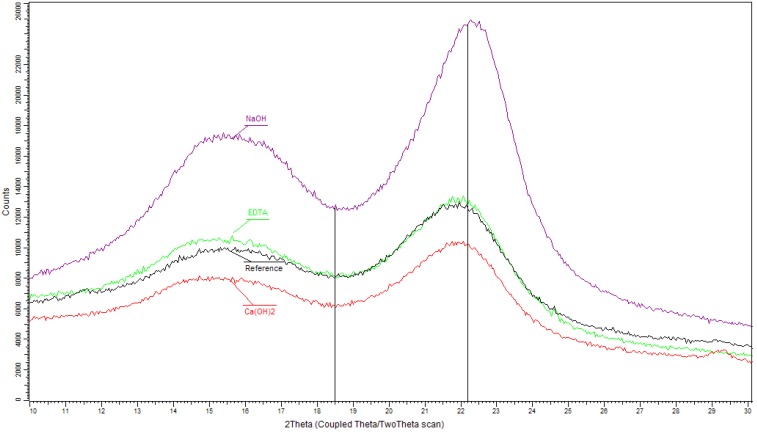
XRD patterns of hemp hurds samples before (referential) and after chemical modification with marked peaks for determination of the crystalline cellulose contents.

The crystallinity index of cellulose from XRD patterns was calculated according to the peak height method for native cellulose [27]. Equation (2) after substraction of the background signal without cellulose was used for the calculation of CI_XRD_. As shown in Figure 3, the intensity of the (200) peak at 2θ = 22.2° represents both crystalline and amorphous cellulose while the height of the minimum at 2θ = 18.5° (*I*_110_ = *I*_AM_) corresponds to amorphous phase:

CI_XRD_(%) = (*I*_200_ − *I*_AM_)/*I*_200_ × 100 (2)

The expression requires diffracting the amorphous material with the same intensity at 18.5° and 22.2°, and the crystalline cellulose does not contribute to the intensity at 18.5° [28].

#### 2.2.5. Size Exclusion Chromatography

Molecular weight distribution analysis of the cellulose samples was performed by size exclusion chromatography (SEC) after their conversion into tricarbanilates. Cellulose tricarbanilates were dissolved in tetrahydrofuran and filtered through a Puradisc 25 NYL filter (Whatman International, Maidstone, UK) with a pore size of 0.45 μm. SEC was performed at 35 °C with tetrahydrofuran at a flow rate of 1 mL·min^−1^ on two PLgel (porous polystyrene/divinylbenzene matrix with particle size of 10 μm and internal diameter × length of 7.5 × 300 mm) MIXED-B columns (Agilent Technologies, Santa Clara, CA, USA) preceded by a PLgel (10 μm, 7.5 × 50 mm), Guard-column (Agilent Technologies) as described by Kacik *et al*. [29]. Data acquisitions were carried out with ChemStation software (Agilent Technologies) and calculations were performed with the Clarity GPC (Gel Permeation Chromatography) module (DataApex, Prague, Czech Republic). Numerical outputs obtained for *M*_n_ (number-average molecular weight) and *M*_w_ (weight-average molecular weight) were recalculated to underivatized cellulose by multiplication with the coefficient *k* = 162/519. Polydispersity index (PDI) of cellulose was calculated as the ratio *M*_w_/*M*_n_. Degree of polymerization (DP) values were calculated by dividing the molecular weight by the monomer equivalent weight of anhydroglucose (DP_w_ = *M*/162).

#### 2.2.6. Thermal Analysis

Thermal analysis (TG/DSC) of hemp hurds was performed by instrument STA 449F3 (NETZSCH, Selb, Germany). Thermal decomposition of the samples was monitored from 25 to 1000 °C, heating rate was 10 K/min and alumina DSC/TG holder was used.

## 3. Results and Discussion

### 3.1. Surface Morphology

**Figure 4:**
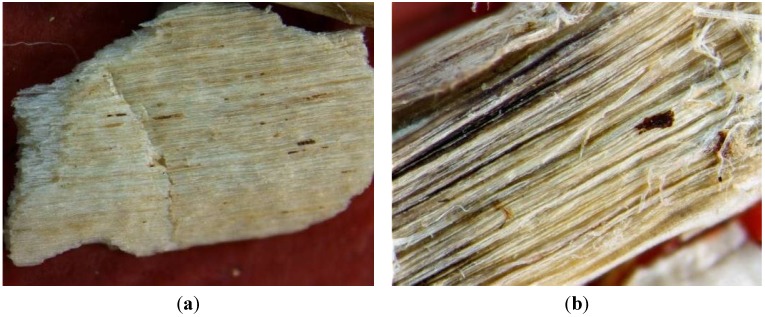

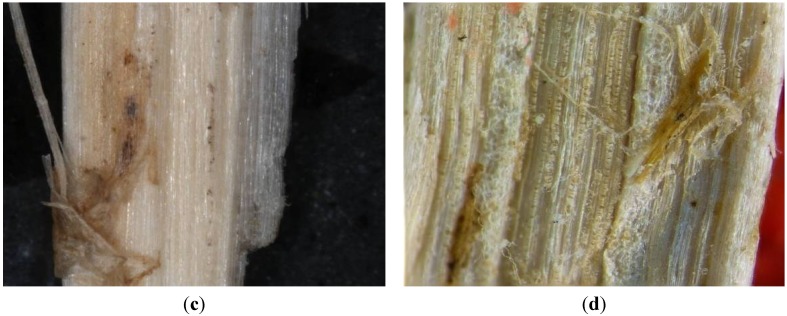
Micrographs of original sample of hemp hurds (**a**) and chemically modified by NaOH (**b**), EDTA (**c**) and Ca(OH)_2_ (**d**) (40 times of magnification).

Changes in structure of hemp fiber bundles were examined by optical microscopy. As can be seen in Figure 4, the chemical modification of hemp hurds led to changes in morphology and structure of fiber bundles in hemp hurds. The photomicrographs of the original samples show presence of dirt and waxes on the surface of hurds. The structure is compact and unfibrilled. The strongest defibrillation of fibrils in hemp hurds sample occurred after treatment with NaOH. The partial separation of fibers there was observed by using of EDTA modification. Hemp hurds treatment with Ca(OH)_2_ led to saturation of calcium ions to the surface of the fibers.

### 3.2. Chemical Composition

Table 4 shows changes in chemical composition of chemically modified hemp hurds in comparison with the original sample. The use of the modification of hurds in the environment of NaOH is well-known to remove amorphous materials, such as hemicelluloses and pectins, from the surface of hemp fibers [30].

By comparing the main components of hemp hurd samples in Table 4, some interesting observations have been found. The hemicellulose percentage is significantly lower in the case of hemp hurds modified in NaOH. By using this agent for modification of hurds, the increase in the content of cellulose and lignin is observed. Decrease in polysaccharides component (holocellulose) is caused mainly by degradation of hemicelluloses. On the other hand, the contents of hemicellulose, cellulose and lignin in samples modified by using EDTA and Ca(OH)_2_ did not exhibit differences in comparison to original hemp hurds. A reduction of lipophilic extractives and ash in all treated samples in comparison with referential sample was measured.

**Table 4:** Comparison of changes in contents of hemp hurd components (%) before and after chemical treatment.

Hemp hurds component	Original sample	Sample modified by		
		NaOH	EDTA	Ca(OH)_2_
Toluene-ethanol extract	3.57	2.8	2.9	2.49
Holocellulose	77.28	65.93	76.75	74.63
Lignin	21.03	27.27	24.22	23.98
Celullose	44.5	53.87	45.7	45.75
Hemicellulose	32.78	12.06	31.05	28.88
Ash	3.04	1.17	1.03	1.39

### 3.3. Structural Changes

**Figure 5:**
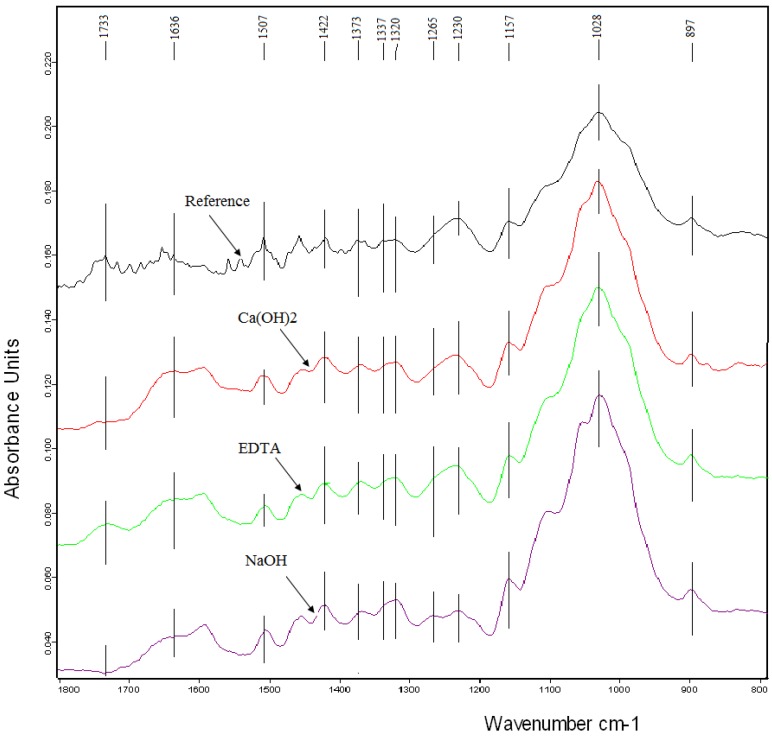
FTIR spectra of reference hemp hurds and chemically modified samples.

**Figure 6:**
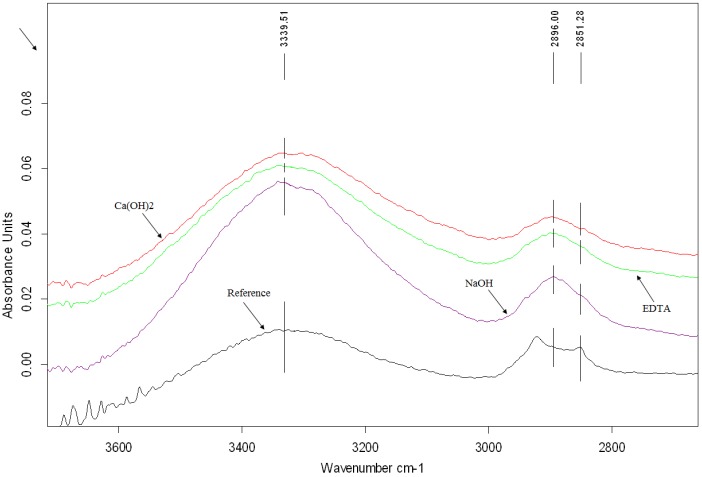
FTIR spectra of reference hemp hurds and chemically modified samples.

FTIR spectroscopy is the most useful in providing information about molecular fragments, the presence or absence of specific functional groups. This method is often used to assess of the degradation level of wood samples after chemical, thermal and weathering processes [29,31]. The changes in FTIR spectra due to chemical treatment of hemp hurds are shown in two regions between 1800 and 700 cm^−1^ and 3300–2700 cm^−1^, respectively, in Figure 5 and Figure 6. The major bands observed in the FTIR spectra of hemp hurds and their assignments to vibrations of chemical group and molecules are summarized in Table 5. Peak positions are consistent with the literature data published for the wood samples as well as vegetable fibers [32,33,34]. There are presented and discussed only the most visible differences in the spectra of chemically treated samples and reference sample of hemp hurds. One such is the modification of the signal at 1733 cm^−1^, characteristic for the stretching vibration of an unconjugated carbonyl group present in hemicelluloses (Figure 5). This peak has fully disappeared after treatment with NaOH and has a very weak intensity after Ca(OH)_2_ treatment. Indeed, treatments with lime water and NaOH, are known to remove hemicelluloses [35]. The observation of the peak located at 1507 cm^−1^ after all treatments seems to indicate that none of the chemical treatment is successful in completely removing lignin from hemp fiber bundles. It corresponds to literature [36,37], according to which lignin cannot be totally removed by the alkaline process. This fact consists in the complicated process of lignin degradation or fragmentation due to the presence of strong C–C linkages and other very resistant function groups such as aromatic groups.

Typical bands assigned to cellulose were observed at 896 cm^−1^ and in the region of 1630–1160 cm^−1^ (Figure 5). The band at 896 cm^−1^ belongs to vibration of glycosidic bonds in polysaccharides. The intensity of the peak located at 1636 cm^−1^ corresponding to water absorbed in cellulose increases slightly after treatment with NaOH. In accordance with papers [38], NaOH reacts with hydroxyl groups present in cellulose forming the water molecules.

In spectra recorded for all treated samples, the intensity of the peak characteristic for waxes and oils present at 2851 cm^−1^ disappears (Figure 6). For the hemp hurds sample treated in NaOH, the peak intensity characteristic for polysaccharides hydroxyl bonds located near 3300 cm^−1^ is sharper.

**Table 5:** Main function groups observed in FTIR spectra of hemp hurds samples (A: amorphous; C: crystalline).

Wavenumber (cm^−1^)	Vibration of function group	Source
3340	OH stretching	polysaccharides
2897	C–H symmetrical stretching	polysaccharides
1733	C=O unconjugated stretching	hemicellulose
1636	OH (water)	cellulose
1507	C=C symmetrical stretching of the aromatic ring	lignin
1454	CH_2_ bending	lignin
1422	CH_2_ bending	cellulose
1373	CH bending	cellulose
1337	OH in plane bending	cellulose (A)
1320	CH_2_ wagging	cellulose (C)
1265	CO stretching	lignin
1157	C–O–C asymmetric bridge oxygen stretching	cellulose
1028	C–C, C–OH, C–H ring and side group vibrations	hemicellulose, pectin
896	glycosidic bonds symmetric ring-stretching mode	polysaccharides

### 3.4. Changes in Cellulose Crystallinity

Cellulose as an important polysaccharide component of hemp (insoluble in water) is a long polymer built up of a great many d-anhydroglucopyranose units joined by β-1,4-glycosidic bonds. The very easily formed intra- and intermolecular hydrogen bonds allow cellulose to function as a structural polymer occurring in various ordered crystalline arrangements. All native celluloses in the higher plants have the crystal structure of cellulose I that is a mixture of triclinic and monoclinic form in ordered and amorphous phase. Based on the resulting structure, the degree of crystallinity and the type of lattice different properties of natural cellulose are observed. In the cellulosic materials, crystalline areas are widely scattered randomly in a continuous manner along the fiber and the relative content of cellulose crystalline phase is usually quantified as the crystallinity index (CI) [39]. The CI of various types of celluloses has been determined by using a variety of methods relying on different structural features. FTIR spectroscopy and X-ray diffraction analysis as the most used for determination of the CI cellulose were selected. Table 6 shows the results of the cellulose crystallinity index for hemp hurd samples before and after chemical treatments which were calculated by Equations (1) and (2) from the data of both used methods. Comparing the proportion of crystalline cellulose in all hemp hurds determined by both methods confirms the NaOH modification of hemp hurds has the greatest impact on increasing CI due to a partial removal of amorphous components of lignin and hemicelluloses. According to the literature [40], this increase in the crystallinity of cellulose during alkaline treatment of hemp hurds can be connected with a loss of the less ordered cellulose too. The apparent increase in the crystalline fraction of cellulose observed in the wood and hemp samples is in agreement with data reported by Lionetto *et al.* and Kostic *et al*. [31,36]. The comparison of the ratios of the cellulose crystallinity of modified and referential hemp hurds samples shows the sequence of the effectiveness of the reagents: NaOH > Ca(OH)_2_ > EDTA.

As is evident from data given in Table 6, differences in the numerical values of crystallinity index determined by both methods are observed and these values cannot be directly comparable. However, correlation between the content values of crystalline cellulose calculated from FTIR and XRD measurements is very good (*R*^2^ = 0.9647).

**Table 6:** Comparison of crystallinity index values CI_FTIR_ and CI_XRD_ for hemp hurd samples before and after chemical treatment.

Hemp hurds sample	Crystallinity index (%)		Crystallinity index ratio	
	CI_FTIR_	CI_XRD_	FTIR	XRD
Referential	55.6	35.7	-	-
NaOH modified	90.2	49.2	1.62	1.39
EDTA modified	62.5	36.6	1.16	1.03
Ca(OH)_2_ modified	87.5	39.8	1.57	1.12

Measurements of cellulose crystallinity content have long history, it has been found that CI varies significantly depending on the choice of measurement method, data evaluation procedure applied to the measurement and on the perfection of the sample [41]. There are several reasons for clarifying the differences in cellulose crystallinity results. FTIR spectroscopy as the simplest method seems to be more suitable for cellulose crystallinity determination in hemp hurds, even though the spectrum may contain contributions from both crystalline and amorphous regions. The determination of cellulose crystallinity in natural material by XRD method is influenced by larger number of factors which can be not eliminated. Most importantly the cellulose crystallites are so small that peak overlapping is bound to occur in the region around 18° for monoclinic cellulose giving too high values of *I*_AM_. The fact that *I*_200_ was overestimated due to preferred orientation of the crystallites added another element of uncertainty to the system. When comparing diffraction patterns for cellulose samples originating from different species there is also the effect that both the apparent cell parameters and the crystallite sizes vary considerably from species to species and influences the relative intensities of peaks. The apparent changes in CI and crystallite size may be due to a re-crystallization of semicrystalline cellulose after the removal of lignin and hemicellulose [42]. Since the XRD is a simple and most popular method for estimating cellulose CI, it has been extensively utilized up to this day [43,44] despite the mentioned limitations and unreliability [45]. Based on the presented results and in accordance with conclusion in paper [46], the FTIR method is more useful for CI evaluation in natural lignocellulosic material. This relative measurement technique giving only relative values is sufficient for comparing the relative differences between hemp hurd samples before and after modification in terms of their utilization in composites.

### 3.5. Changes in Degree of Cellulose Polymerization

Structure of cellulose as an important hemp polymer component contains both a crystalline and amorphous phase. The attention is being focusing on determining the important structural parameter—degree of polymerization (DP) of cellulose that varies according to its sources, acquiring processes and further treatments. Structural changes in cellulose having a significant effect on the mechanical properties of hemp hurds were studied by the method of size exclusion chromatography (SEC). This method allows the determination of the average cellulose chain length, measured as degree of polymerization (DP). As is shown in Table 7, all studied chemical treatment caused reduction in DP values of cellulose polymerization what is related to the cleavage of glycosidic bonds and shortening cellulose chains.

**Table 7:** The values of number-average molecular weight (*M*_n_), weight-average molecular weight (*M*_w_), polydispersity index (PDI) of cellulose and degree of polymerization of cellulose (DP) in reference sample and treated hemp hurds.

Hemp hurds sample	*M*_w_	*M*_n_	PDI	DP
Reference	210,934	18,624	11.33	1,302
NaOH modified	94,851	15,469	6.13	585
EDTA modified	150,436	14,712	10.23	929
Ca(OH)_2_ modified	141,175	15,139	9.33	871

**Figure 7:**
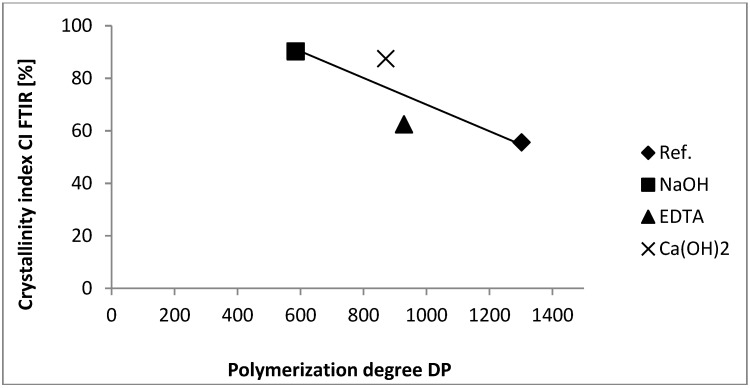
Relationship between DP values and CI_FTIR_ of cellulose.

The most significant decrease in DP value in comparison to referential sample is observed for the hemp hurds sample modified in NaOH solution. Figure 7 shows a strong correlation (*R*^2^ = −0.885) between the obtained DP values and the CI_FTIR_ of cellulose in hemp hurd samples. It is clear that cellulose CI decreases with increasing degree of polymerization.

**Figure 8:**
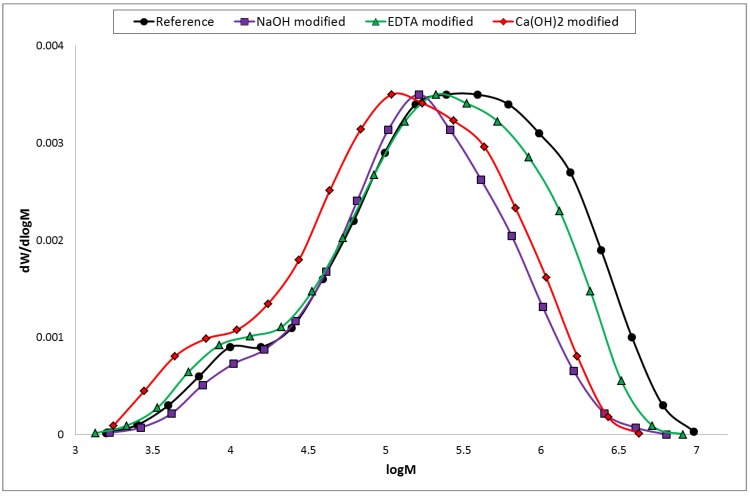
Cellulose molecular weight distribution of hemp hurds samples.

Molecular weight distributions (MWD) of the cellulose samples constructed from chromatograms are shown in Figure 8 and polydispersity index (PDI) values are in Table 7. As it can be seen, the value of PDI decreases from 11.33 (reference sample) to 6.13 (NaOH modified sample). This fact is connected with the degradation of high molecular weight fractions (Figure 8).

### 3.6. Water Absorbability

As is seen in Table 8, measurements of water contents in chemically treated hemp hurd samples showed a significant decrease of water amount when compared to reference (untreated) sample.

**Table 8:** The values of water content in reference sample and in chemically treated hemp hurds.

Water content (%)			
Reference sample	Sample modified by		
	NaOH	EDTA	Ca(OH)_2_
10.78	3.11	6.99	6.67

According to published data [47], the hydrophilic behavior of plant fibers depends on their composition and specific structure. Based on knowledge of the water absorption in the cellulose structure [48] and our findings, water absorbability of treated samples is related to crystallinity degree of the cellulose. The water content decreases as the crystallinity degree of the cellulose increases in chemically treated hemp hurds samples. Amorphous component such as hemicellulose plays also an important role in the water storage. Significant lower water content was observed in hemp fibers after removal of hemicelluloses and lignin [49]. The positive influence of chemical treatment of hemp hurds on the absorption behavior of composites based on modified filler during their long-term storage in water was observed [50].

### 3.7. Thermal Stability

The study of thermal stability of hemp hurds is very important due to its subsequent incorporation into composite materials and use of the products thus prepared for various construction purposes.

**Figure 9:**
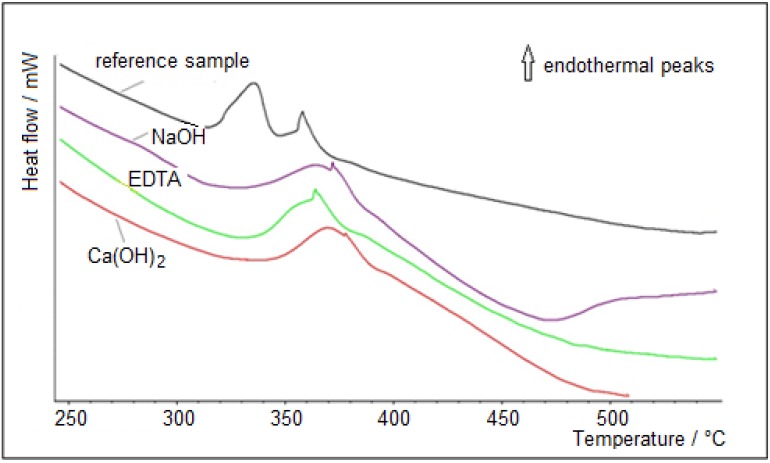
DSC curves of referential and chemically treated hemp hurds.

Figure 9 shows details of DSC curves of hemp hurds in the most important temperature range of 250–500 °C. Degradation processes occurring in three temperature ranges during heating are specified in Table 9. As seen from the DSC results, curves for modified hurds have a different course in depending on the used agents compared to the referential sample. In case of all treated samples, the onset of temperature degradation of the components of hemp hurds is observed at higher temperatures in comparison to the original sample. However, the interpretation of DSC measurements of cellulosic materials is complicated by the decomposition over the whole temperature range (280–500 °C) of lignin residues. In all cases of treatment of hemp hurds, degradation of cellulose is difficult to disentangle on the DDSC (derived differential scanning calorimetry) curves because the peaks overlap. We were able to determine only the peak top but not its whole course. These findings are in accordance with data from the literature [51].

Cellulose is more resistant to thermal treatment in comparison to hemicellulose probably due to its crystalline structure. When heated cellulose ongoing three primary reactions: oxidation, dehydration and depolymerization. Thermooxidation and dehydration taking place mainly in the amorphous phase of polysaccharides. At temperatures above 300 °C, depolymerization becomes the dominant reaction of the degradation of cellulose. Top of peaks of cellulose decomposition was recorded about 358–378 °C. These findings on the hemicellulose and cellulose decomposition are in good agreement with literature data [15]. As it is evident from Table 9, lignin degradation proceeds gradually in three temperature ranges. The highest value of total weight loss is 92.89 wt% for original sample.

**Table 9:** Temperature ranges of hemp hurd components degradation and weight loss.

Sample	Temperature ranges of components degradation (ºC)			
	1. peak	2. peak	3. peak	Weight loss (%)
Referential	320–335 °C	358 °C	311–384 °C	92.89
NaOH	320–370 °C	371 °C	320–385 °C	90.92
EDTA	330–358 °C	364 °C	330–390 °C	91.05
Ca(OH)_2_	337–377 °C	378 °C	349–390 °C	90.40
	Depolymerization of hemicellulose and pectin + degradation of lignin	Decomposition of cellulose + degradation of lignin	Degradation of lignin	-

## 4. Conclusions

Experiments were conducted to characterize the changes in the properties of hemp hurds after its chemical modification in three solutions (NaOH, EDTA and Ca(OH)_2_) by using physic-chemical methods in terms of the potential use of this natural material as filler in composite materials.

The most significant changes in defibrillation of bundles, chemical and phase composition and cellulose crystallinity were recorded in the case of alkaline treated hemp hurds sample. Decrease in lignin and hemicellulose content was obtained. Content of polysaccharides component (holocellulose) was lower in comparison to referential sample due to degradation of hemicelluloses.

The highest value of crystallinity index of cellulose calculated from both FTIR and XRD methods was detected after NaOH treatment of hemp hurds. The results of CI_FTIR_ and CI_XRD_ are in a strong correlation. Based on the presented results and in accordance with literature, FTIR method seems be more useful for crystallinity evaluation of cellulose in natural lignocellulosic material.

Chemical treatment led to decrease in degree of polymerization of cellulose. A good correlation between degree of polymerization and crystallinity index (CI_FTIR_) of cellulose for all hemp hurd samples was obtained. NaOH treatment of hemp hurds led to a significant decrease in polydispersity index in comparison to reference sample due to the degradation of high molecular weight fractions of cellulose. Water absorbability of hemp hurds has been enhanced after chemical treatment. The obtained results confirmed that water behavior depends on chemical composition of hemp hurd samples.

Thermal stability of chemically modified hemp hurd samples was higher than in the case of reference sample.

Such an approach based on knowledge of the changes in morphology, chemical composition and structure of hemp hurds after chemical treatment is very beneficial for the preparation of composite materials of progressively higher performance in terms of improvement of filler/matrix interface properties.

## References

[1] Bakis C.E., Bank L.C., Brown V.L., Cosenza E., Davalos J.F., Lesko J.J., Machida A., Rizkalla S.H., Triantafillou T.C. (2002). Fiber-reinforced polymer composites for construction—A state-of-the-art review. J. Compos. Constr..

[2] Yan L., Chouw N., Lu Y. (2014). Sustainable concrete and structures with natural fibre reinforcements. Infrastructure Corrosion and Durability—A Sustainability Study.

[3] Savastano H., Santos S.F., Radonjic M., Soboyejo W.O. (2009). Fracture and fatigue of natural fiber-reinforced cementitious composites. Cem. Concr. Compos..

[4] Thygesen A., Daniel G., Lilholt H., Thomsen A.B. (2005). Hemp fiber microstructure and use of fungal defibration to obtain fibers for composite materials. J. Nat. Fibres.

[5] Gümüşkaya E., Usta M., Balaban M. (2007). Carbohydrate components and crystalline structure of organosolv hemp (Cannabis sativa L.) bast fibers pulp. Bioresour. Technol..

[6] Thomsen A.B., Rasmussen S., Bohn V., Vad Nielsen K., Thygesen A. (2005). Hemp Raw Materials: The Effect of Cultivar, Growth Conditions and Pretreatment on the Chemical Composition of the Fibres.

[7] Angelini L.G., Lazzeri A., Levita G., Fontanelli D., Bozzi C. (2000). Ramie (Boehmeria nivea (L.) Gaud.) and Spanish Broom (Spartium junceum L.) fibres for composite materials: Agronomical aspects, morphology and mechanical properties. Ind. Crops Prod..

[8] Madsen B., Hoffmeyer P., Thomason A.B., Lilhot H. (2007). Hemp yarn reinforced composites—I. Yarn characteristics. Compos. A Manufact..

[9] Preikss I., Skujans J., Adamovics A., Iljins U. (2013). Evaluation of hemp (Cannabis Sativa L.) quality parameters for building material from foam gypsum products. Chem. Eng. Trans..

[10] Kidalova L., Stevulova N., Terpakova E., Sicakova A. (2012). Utilization of alternative materials in lightweight composites. J. Clean. Prod..

[11] Kidalova L., Stevulova N., Junak J. Study of strength characteristics of hemp composite. Proceedings of 4th International Conference on Engineering for Waste and Biomass Valorisation.

[12] Cigasova J., Stevulova N., Junak J. (2013). Properties monitoring of fibrous composites based on hemp hurds with different mean particle size. Pollack Period..

[13] Cigasova J., Stevulova N., Sicakova A., Junak J. (2013). Some aspects of lightweight composites durability. Chem. Eng. Trans..

[14] Elfordy S., Lucas F., Tancret F., Scudeller Y., Goudet L. (2008). Mechanical and thermal properties of lime and hemp concrete (“hempcrete”) manufactured by a projection process. Construct. Build. Mater..

[15] Ouajai S., Shanks R.A. (2005). Composition, structure and thermal degradation of hemp cellulose after chemical treatment. Polym. Degrad. Stab..

[16] Mwaikambo L.Y., Ansell M.P. (2002). Chemical modification of hemp, sisal, jute and kapok fibers by alkalization. J. Appl. Poly. Sci..

[17] Thomsen A.B., Thygesen A., Bohn V., Nielsen K.V., Allesen B., Jorgensen M.S. (2006). Effects of chemical and physical pre-treatment processes on hemp fibres for reinforcement of composites and for textiles. Ind. Crop. Prod..

[18] Renouard S., Hano C., Doussot J., Blondeau J.P., Lainé E. (2014). Characterization of ultrasonic impact on coir, flax and hemp fibers. Mater. Lett..

[19] Terpakova E., Kidalova L., Estokova A., Cigasova J., Stevulova N. (2012). Chemical modification of hemp shives and their characterization. Procedia Eng..

[20] Stevulova N., Cigasova J., Sicakova A., Junak J. (2013). Lightweight composites based on rapidly renewable natural resource. Chem. Eng. Trans..

[21] (1998). Standard Test Method for Ethanol Toluene Solubility of Wood.

[22] Wise L.E., Maxine M., D’Addieco A.A. (1946). Chlorite holoeellulose, its fractionation and bearing on summative wood analysis and on studies on the hemicelluloses. Paper Trade J..

[23] Seifert V.K. (1956). Über ein neues Verfahren zur Schnellbestimmung der Rein—Cellulose. Papier.

[24] Sluiter A., Hames B., Ruiz R., Scarlata C., Sluiter J., Templeton D., Crocker D. (2010). Determination of Structural Carbohydrates and Lignin in Biomass.

[25] Sluiter A., Hames B., Ruiz R., Scarlata C., Sluiter J., Templeton D. (2008). Determination of Ash in Biomass.

[26] Le Troëdec M., Sedan D., Peyratout C., Bonnet J.P., Smith A., Guinebretiere R., Gloaguen V., Krausz P. (2008). Influence of various chemical treatments on the composition and structure of hemp fibres. Compos. A Manufact..

[27] Dai D., Fan M. (2010). Characteristic and performance of elementary hemp fibre. Mater. Sci. Appl..

[28] Segal L., Creely J.J., Martin A.E., Conrad C.M. (1962). An empirical method for estimating the degree of crystallinity of native cellulose using the X-ray diffractometer. Text. Res. J..

[29] Kacik F., Kacikova D., Jablonsky M., Katuscak S. (2009). Cellulose degradation in new-sprint paper ageing. Polym. Degrad. Stab..

[30] Le Troëdec M., Rachini A., Peyratout C., Rossignol S., Maxb E., Kaftan O., Fery A., Smith A. (2011). Influence of chemical treatments on adhesion properties of hemp fibres. J. Colloid Interface Sci..

[31] Lionetto F., Del Sole R., Cannoletta D., Vasapollo G., Maffezzoli A. (2012). Monitoring wood degradation during weathering by cellulose crystallinity. Materials.

[32] Le Troëdec M., Peyratout C.S., Smith A., Chotard T. (2009). Influence of various chemical treatments on the interactions between hemp fibres and a lime matrix. J. Eur. Ceram. Soc..

[33] Sawpan M.A., Pickering K.L., Fernyhough A. (2011). Effect of various chemical treatments on the fiber structure and tensile properties of industrial hemp fibres. Compos. A Manufact..

[34] Liu Y. (2013). Recent progression fourier transform infrared (FTIR) spectroscopystudy of compositional, structure and physical attributes of developmental cotton fibers. Materials.

[35] Zhang W., Okubayashi S., Bechtold T. (2005). Fibrillation of cellulosic fibers—Part 4: Effects of alkali pre-treatment of various cellulosic fibers. Carbohydr. Polym..

[36] Kostic M., Pejic B., Scundric P. (2008). Quality of chemically modified hemp fibers. Bioresour. Technol..

[37] Wang H.M., Postele R., Kessler R., Kessler W. (2003). Removing pectin and lignin during chemical processing of hemp for textile applications. Text. Res. J..

[38] Agrawal R., Saxena N.S., Sharma K.B., Thomas S., Sreekala M.S. (2000). Activation energy and crystallization kinetics of untreated and treated oil palm fibre reinforced phenol formaldehyde composites. Mater. Sci. Eng. A.

[39] Gumuskaya E., Usta M. (2006). Dependence of chemical and crystalline structure of alkali sulfite pulp on cooking temperature and time. Carbohydr. Polym..

[40] Evans R., Newman R.H., Roick U.C., Suckling I.D., Wallis F.A. (1995). Changes in cellulose crystallinity during kraft pulping. Comparison of infrared, X-ray diffraction and solid state NMR result. Holzforschung.

[41] Park S., Baker J.O., Himmel M.E., Parilla P.A., Johnson D.K. (2010). Research cellulose crystallinity index: Measurement techniques and their impact on interpreting cellulose performance. Biotechnol. Biofuels.

[42] Howell C., Steenkjaer Hastrup A.C., Goodell B., Jellison J. (2009). Temporal changes in wood crystalline cellulose during degradation by brown rot fungi. Int. Biodeterior. Biodegrad..

[43] Mwaikambo L.Y., Ansell M.P. (1999). The effect of chemical treatment on the properties of hemp, sisal, jute, and kapok for composite reinforcement. Angew. Makromol. Chem..

[44] De Souza I.J., Bouchard J., Method M., Berry R., Argyropoulos D.S. (2002). Carbohydrates in oxygen delignification. Part I: Changes in cellulose crystallinity. J. Pulp Paper Sci..

[45] Thygesen A., Oddershede J., Lilholt H., Thomsen A.B., Ståhl K. (2005). On the determination of crystallinity and cellulose content in plant fibres. Cellulose.

[46] Fan M., Dai D., Huang B., Salih S.M. (2012). Fourier transform infrared spectroscopy for natural fibres. Fourier Transform—Materials Analysis.

[47] Nakamura K., Hatakeyama T., Hatakeyama H. (1981). Studies of bound water cellulose by differential scanning calorimetry. Text. Res. J..

[48] Célino A., Freór S., Jacquemin F., Casari P. (2014). The hygroscopic behaviour of plant fibres: A review. Front. Chem..

[49] Pejic B.M., Kostic M.M., Skundric P.D., Praskalo J.Z. (2008). The effects of hemicelluloses and lignin removal on water uptake behaviour of hemp fibres. Bioresour. Technol..

[50] Stevulova N., Schwarzova I. (2014). Changes in the properties of composites caused by chemical treatment of hemp hurds. Int. J. Chem. Nucl. Metall. Mater. Eng..

[51] Lu N., Shubhashini O. (2013). Thermal stability and thermo-mechanical properties of hemp-high density polyethylene composites: Effect of two different chemical modifications. Compos. B Eng..

